# Persisting Vaccine Hesitancy in Africa: The Whys, Global Public Health Consequences and Ways-Out—COVID-19 Vaccination Acceptance Rates as Case-in-Point

**DOI:** 10.3390/vaccines10111934

**Published:** 2022-11-15

**Authors:** Emmanuel O. Njoga, Olajoju J. Awoyomi, Onyinye S. Onwumere-Idolor, Priscilla O. Awoyomi, Iniobong C. I. Ugochukwu, Stella N. Ozioko

**Affiliations:** 1 Department of Veterinary Public Health and Preventive Medicine, Faculty of Veterinary Medicine, University of Nigeria, Nsukka 410001, Nigeria; 2 Department of Veterinary Public Health and Preventive Medicine, College of Veterinary Medicine, Federal University of Agriculture, Abeokuta PMB 2240, Nigeria; 3 Department of Animal Production, Faculty of Agriculture, Delta State University of Science and Technology, Ozoro PMB 005, Nigeria; 4 Department of Medicine and Surgery, College of Medicine, University of Ibadan, Ibadan 200005, Nigeria; 5 Department of Veterinary Pathology and Microbiology, Faculty of Veterinary Medicine, University of Nigeria, Nsukka 410001, Nigeria; 6 Department of Veterinary Medicine, Faculty of Veterinary Medicine, Universita degli Studi di Bari, 70010 Valenzano, Italy; 7 Institute of Aquaculture, Faculty of Natural Sciences, University of Stirling, Stirling FK9 4LA, UK

**Keywords:** Africa, COVID-19 vaccine acceptance rate, global public health, SARS-CoV-2, vaccine hesitance, vaccine inequality, ways-out

## Abstract

Vaccine hesitancy (VH) is the seventh among the WHO’s top 10 threats to global public health, which has continued to perpetuate the transmission of vaccine preventable diseases (VPDs) in Africa. Consequently, this paper systematically reviewed COVID-19 vaccine acceptance rates (VARs)—including the vaccine uptake and vaccination intention—in Africa from 2020 to 2022, compared the rates within the five African regions and determined the context-specific causes of VH in Africa. Generally, COVID-19 VARs ranged from 21.0% to 97.9% and 8.2% to 92.0% with mean rates of 59.8 ± 3.8% and 58.0 ± 2.4% in 2021 and 2022, respectively. Southern and eastern African regions had the top two VARs of 83.5 ± 6.3% and 68.9 ± 6.6% in 2021, and 64.2 ± 4.6% and 61.2 ± 5.1% in 2022, respectively. Based on population types, healthcare workers had a marginal increase in their mean COVID-19 VARs from 55.5 ± 5.6% in 2021 to 60.8 ± 5.3% in 2022. In other populations, the mean VARs decreased from 62.7 ± 5.2% in 2021 to 54.5 ± 4% in 2022. As of 25 October 2022, Africa lags behind the world with only 24% full COVID-19 vaccinations compared to 84%, 79% and 63% reported, respectively, in the Australian continent, upper-middle-income countries and globally. Apart from the problems of confidence, complacency, convenience, communications and context, the context-specific factors driving COVID-19 VH in Africa are global COVID-19 vaccine inequality, lack of vaccine production/maintenance facilities, insecurity, high illiteracy level, endemic corruption, mistrust in some political leaders, the spreading of unconfirmed anti-vaccination rumors and political instability. With an overall mean COVID-19 acceptance rate of 58%, VH still subsists in Africa. The low VARs in Africa have detrimental global public health implications, as it could facilitate the emergence of immune invading SARS-CoV-2 variants of concern, which may spread globally. Consequently, there is a need to confront these challenges frontally and engage traditional and religious leaders in the fight against VH in Africa, to restore public trust in the safety and efficacy of vaccines generally. As the availability of COVID-19 vaccines improves, the vaccination of pets and zoo-animals from which reverse zoonotic transmission of SARS-CoV-2 have been reported is recommended, to limit the evolution and spread of new variants of concern and avert possible SARS-CoV-2 epizootic or panzootic diseases in susceptible animal species.

## 1. Introduction

Since 1796, when Edward Jenner accidentally discovered that inoculation with cowpox virus conferred cross-immunity to smallpox disease, vaccination has remained the bedrock of preventive medicine in both medical and veterinary practices. Annually, vaccination against vaccine-preventable diseases (VPDs) averts three million deaths worldwide and an additional 1.5 million lives could be saved if global vaccination coverage improves [1]. The smallpox and rinderpest have been eradicated globally through vaccination, while a whole lot of others (polio, tetanus, whooping cough, measles, rubella, etc.) have at least been largely controlled [2]. Recent advancements in the field of vaccinology, such as the production of DNA sub-unit vaccines and mRNA technology, are expected to further enhance the success story of vaccination in the prevention and control of VPDs. Regrettably, vaccine hesitancy (VH), which refers to apathy towards, deferral of or outright rejection of vaccines regardless of the availability and accessibility of the vaccination services, has continued to perpetuate the transmission of VPDs to the detriment of global public health especially in rural African settings [3,4]. One of the VPDs that has persisted, probably due to VH, is the coronavirus disease-2019 (COVID-19).

Globally, the COVID-19 pandemic caused by severe acute respiratory syndrome coronavirus-2 (SARS-CoV-2) has infected 633 million people (PCR-confirmed cases reported) of which 6.6 million have died as of 25 October 2022 [5]. This represents a global reported case fatality ratio (CFR) of 1.1% as against 7.2% documented in April 2020 [6]. The decrease in the low CFR may be due to some public health measures against the virus such as hand hygiene, social distancing, vaccination and better patient management in hospitals, including the use of dexamethasone [7]. In Africa, the low CFR compared to other continents was attributed to the early implementation of lockdown and border closures, the predominant younger age population in the continent, underreporting of COVID-19 fatalities and some yet to be identified genetic advantages among people of African origin [8]. Five SARS-CoV-2 variants of concern (VOCs)—Alpha (B.1.1.7), Beta (B.1.351), Gamma (P.1), Delta (B.1.617.2) and Omicron (B.1.1.529)—have emerged since the virus was first reported in 2019 [9]. However, only the Omicron variant and its sub-variants—BA.4, BA.5, BA.2.12.1 and BA.2.75—currently drive the pandemic as of 25 October 2022 [9]. Genetic mutations and deletions at the receptor-binding domain (RBD) and the *N*-terminal region of SARS-CoV-2 spike proteins modify the biology of the virus, and enhance its transmissibility by increasing the affinity of the spike proteins for mammalian angiotensin-converting enzyme-2 (ACE-2) [10,11]. These changes may also affect the immunogenicity of the virus and enhance the immune evasion potentials. These genetic alterations make host cell recognition of VOCs difficult and immune escape inevitable, as the SARS-CoV-2-neutralizing antibodies (produced after vaccination or recovery from COVID-19) are specific and may not bind to the RBD of a different variant [10,11]. Consequently, some COVID-19 vaccines may not prevent the severe form of the disease following infection with new VOCs even in fully vaccinated individuals.

In response, to control the deadly pandemic and alleviate the resultant untoward impacts on global health, the economy and social life, some COVID-19 vaccines were swiftly produced, approved by various world/regional regulatory bodies and then rolled-out. Unarguably, mass vaccination is the best approach for controlling infectious disease outbreaks, especially those that have attained a pandemic status [12]. Some COVID-19 vaccines have been proven to be safe and efficacious in reducing severe illness and death. The Cuban protein sub-unit vaccine, Abdala, demonstrated safety, tolerability and efficacy (92.3% [95% CI: 85.7–95.8]) against SARS-CoV-2 in clinical trials in Havana, Cuba [13]. In a recent nation-wide study to test the efficacy of COVID-19 vaccines among the Greek population, two doses of BNT162b2, mRNA-1273 or ChAdOx1 nCov-19 vaccines offered very high (>90%) vaccine efficacy against both intubation and death across all age groups while a three-dose vaccination protocol increased the efficacy to almost 100% [14]. Although the vaccines’ efficacy waned overtime, they remained >80% protective within the first six months post vaccination, prevented an estimated 19,691 COVID-19 deaths (95% confidence interval: 18,890–20,788), and offered strong and durable protection against COVID-19 severe disease and death [14]. Likewise, in another study conducted by Mohammed et al. [15], the researchers concluded that COVID-19 vaccines have successfully reduced the rates of infections, disease severity, hospitalization and mortality among different populations; and that a full-dose regimen of the Pfizer/BioNTech vaccine is the most effective against infections with the B.1.1.7 and B.1.351 variants.

Globally, 13 billion doses of COVID-19 vaccines have been administered with 5.4% and 68.4% of the population being partly and fully vaccinated as of 25 October 2022, respectively [16]. Full COVID-19 vaccination implies receiving at least two doses of the vaccine or having one dose of a two-dose protocol post natural infection with SARS-CoV-2 [16]. At the continental level, 86.6%, 85.6%, 76.7%, 75%, 69% and 30% of the population have had at least one dose of a COVID-19 vaccine in Australia, South America, Asia, North America, Europe and Africa, respectively [16]. Only 23.3% of people in low-income countries have received at least one dose of a COVID-19 vaccine as of 25 October 2022 [16].

According to the WHO, VH is the seventh among the top 10 threats to global health. Vaccination apathy and refusal is currently frustrating efforts towards the elimination of VPDs in Africa, particularly SARS-CoV-2, and the consequent eradication worldwide [17,18]. Therefore, there is a need to highlight Africa’s peculiar possible causes of VH and recommend feasible solutions to improve COVID-19 vaccine acceptance (including the vaccine uptake and vaccination intention) and coverage in the continent. This has become imperative in view of the low COVID-19 vaccination rates (VARs) in low-income countries, most of which are African countries [19]. Therefore, this paper determined COVID-19 VARs in Africa from 2020 to 2022, and compared the regional and yearly trends in the vaccine acceptance rates as well as other epidemiological variables associated with VH. The paper also compared the COVID-19 VARs across various continents and according to the World Bank’s classification on income levels. Finally, the continent’s specific causes of VH and its deleterious health impacts were discussed and ways-out recommend for global public health safety.

## 2. Materials and Methods

### 2.1. The Study Area

**Figure 1:**
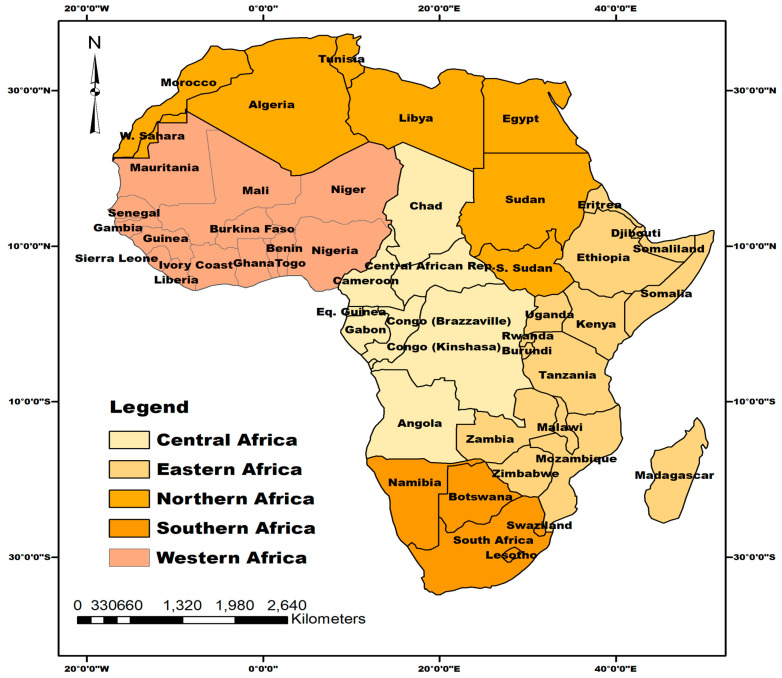
Map of Africa showing the five African regions and their constituent countries where published articles reviewed in this study were selected.

Located between latitude 9.1021° N and longitude 18.2812° E, Africa is the world’s second-largest and second-most-populated continent apart from Asia. The continent is bounded by the Mediterranean Sea to the north, the Isthmus of Suez and the Red Sea to the northeast, the Indian Ocean to the southeast and then the Atlantic Ocean in the west. Africa comprises 55 countries (including Morocco) and five regions (Central, Eastern, Northern, Southern and Western Africa) according to the United Nations’ Geoscheme classification [20], as shown in Figure 1. The continent has an estimated population of 1.42 billion people as of 25 September 2022, representing 16.7% of the global population [21]. Eastern and Western Africa were the top two most-populated regions with estimated populations of 445 and 402 million people, respectively [21]. Africa’s total land mass is 29,648,481 km^2^ with a population density of 45 persons per km^2^ [21]. A total of 40% of the population live in urban areas [21]. The continent has a predominantly youthful population with a median age of 19.7 years [21].

### 2.2. Literature Search

The Preferred Reporting Items for Systematic Reviews and Meta-Analyses (PRISMA) guidelines, as described by Page et al. [22], were strictly followed in this review. Published papers in Scopus, PubMed/Medline, Web of Science and African Journals Online (AJOL) that evaluated COVID-19 vaccine hesitancy and vaccine acceptance (including the vaccine uptake, intention or willing to get vaccinated) based on questionnaire surveys in African countries were reviewed. To be eligible for inclusion in this study, the paper must have been peer-reviewed, published in the English language between 1 January 2020 and 5 September 2022 and have determined COVID-19 VARs in any African country. The search for published literature was conducted as of 5 September 2022, using the following keywords: “COVID-19 vaccine uptake”, “COVID-19 vaccine acceptance”, “COVID-19 vaccine hesitancy”, “COVID-19 vaccination intention”, “Africa” “COVID-19 vaccine acceptance rate” survey. The search strategy also involved Boolean operators and MeSH terms including (“COVID-19” OR “COVID-19” [MeSH Terms] OR “COVID-19 Vaccines” OR “COVID-19 Vaccines” [MeSH Terms] OR “COVID-19 vaccine hesitancy” OR “COVID-19 vaccine acceptance OR COVID-19 vaccine acceptance” [MeSH Terms] OR “COVID-19 vaccination intention” OR “ COVID-19 vaccination intention [MeSH Terms] OR “COVID-19 vaccine acceptance rate” OR “COVID-19 vaccine acceptance rate” [MeSH Terms] “COVID-19 vaccine acceptance and hesitancy” AND “COVID-19 vaccine acceptance and hesitancy” [MeSH Terms] Coronavirus vaccine in Africa” OR “Coronavirus vaccine in Africa” [MeSH Terms] “COVID-19 not vaccinated” AND “COVID-19 not vaccinated” [MeSH Terms] OR (COVID vaccine accept Africa) AND (2020:2022[pdat]).

Duplicate papers from different search engines were removed before the screening of titles and abstracts was performed. Then, data extraction for the following items was conducted: name of country/countries in which the survey was conducted, the target population (which was later categorized as either healthcare workers—HCWs—or other populations), total number of respondents surveyed and COVID-19 VARs. The categorization of the population into HCWs and other population is because the former are more likely to accept COVID-19 vaccination than the latter [23,24]. Thereafter, each country was then assigned into their respective African regions according to the United Nations’ Geoscheme classification [20]. Individual country data were extracted from articles that determined COVID-19 VARs in more than one African country.

Furthermore, data on the global distribution of COVID-19 infections, fatalities and vaccination status (unvaccinated, partly vaccinated and fully vaccinated) in selected African countries and continents were extracted from the WHO database [21] and OurWorldInData.org [16]. Data on the same variables were extracted and presented in charts according to the World Bank’s countries’ classification on income levels. The extracted data were then compared across continents and the World Bank’s countries’ income levels, to highlight possible discrepancies in COVID-19 vaccination, infection and fatality rates; ascertain the possible causes; and then suggest the ways-out.

### 2.3. Data Analyses

The data retrieved from the papers included in the review were entered into SPSS, version 20 (IBM, Armonk, NY, USA) for statistical analysis. Descriptive statistical analysis was carried out and the results presented in frequencies and means. An independence *t*-test was carried out to test for differences in the means of the acceptance rates of COVID-19 vaccination between the HCWs and other populations. The *p*-value was set at 0.05.

## 3. Results

### 3.1. Literature Review

**Figure 2:**
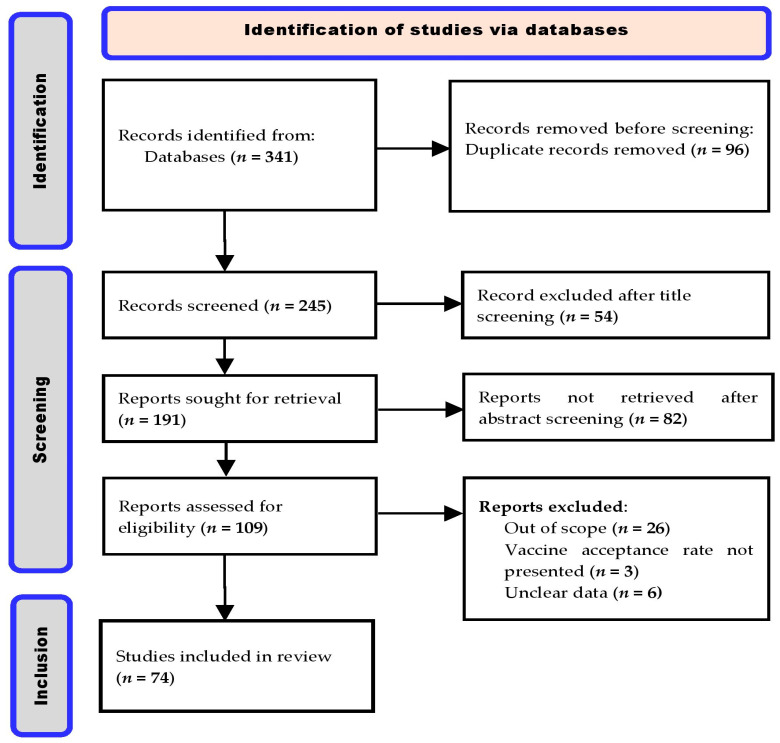
PRISMA flow chart for the search, identification, screening, and inclusion criteria for the 74 published articles reviewed in this study.

A total of 341 peer-reviewed articles were retrieved during the literature search. After the screening process (title and abstract) and the implementation of other eligibility and inclusion criteria, 74 articles that reported COVID-19 VARs were included in this review (Figure 2).

### 3.2. Characteristics of the Papers Included in This Review

The 74 published papers included in this study reported COVID-19 VARs from 20 African countries (Table 1). The distributions of the papers and the various COVID-19 VARs reported, according to countries and regions, are presented in Table 2. The distributions of the articles reviewed, according to the regions, were: Western Africa (*n* = 29), Southern Africa (*n* = 12), Eastern Africa (*n* = 26), Northern Africa (*n* = 12) and Central Africa (*n* = 1). There was no published study on COVID-19 vaccination acceptance in 2020 in any African country; 30 and 44 studies were published in 2021 and 2022, respectively (Table 1). One study reported COVID-19 VARs in multiple (six) African countries while another reported acceptance rates from longitudinal (repeated) surveys in the same country. The number of respondents surveyed ranged between 73 (lowest sample size noted) and 10,465 (largest sample size found). Of the 74 articles reviewed, 29 and 51 reported on COVID-19 VARs among HCWs and other populations, respectively (Table 1). Some of the papers reported COVID-19 VARs in both the HCWs and other populations.

### 3.3. Rates of COVID-19 Vaccine Acceptance Found in the Study

Among the HCWs, high COVID-19 VARs (>90%) were reported in Nigeria and South Africa while low acceptance rates (<20%) were documented in Cameroon, Ethiopia and Nigeria (Table 1). On a regional level, the highest mean COVID-19 VARs of 83.5% and 68.9% were recorded in Southern Africa and Eastern Africa, respectively, while Northern Africa had the lowest mean acceptance of 45.1% in 2021 (Table 2). The regional trend in COVID-19 VARs in 2022 was largely the same, with Southern and Eastern Africa maintaining their leads while Central Africa had the lowest acceptance rate of 13.5% (Table 2).

Among the 29 studies involving HCWs, 10 have less than 50% acceptance rates, and both the highest (92%) and lowest (11.4%) were reported in Nigeria (Table 3). The regions with high COVID-19 VARs among the HCWs were Southern Africa (89.8%) followed by Eastern Africa (54.8%) in 2021 (Table 3). In 2022, eastern African regions have the highest mean VAR (70.5%). However, HCWs in Northern and Western Africa recorded the lowest acceptance rates in 2021 and 2022, respectively (Table 3).

For the non-health workers (other populations), the eastern and southern African regions had higher acceptance rates of 83% and 69.7%, respectively, while the northern African region had 46.6% in 2021 (Table 3). In 2022, southern and western African regions had the top two acceptance rates of 61.9% and 53.7%, respectively. While the overall COVID-19 VARs marginally increased from 55.5% to 60.6% among HCWs in 2022, there was a downward trend in the acceptance rates among non-health workers from 64.4% in 2021 to 55% in 2022 (Table 3). Generally, there was no significant difference (*p* > 0.05) in the overall rate of COVID 19 VARs between HCWs and other populations in Africa.

### 3.4. COVID-19 Infections and Vaccination Rates Extracted from Databases

Data extracted on COVID-19 infections and vaccination rates for the 20 African countries included in this review as of 25 September 2022 and the respective case fatality rates computed are presented as Supplementary Table S1. No African country had attained the minimum 70% full COVID-19 vaccination rate. However, the top three countries that had the highest total (full and partial) vaccination rates were Tunisia (73%), Morocco (67%) and Botswana (58%). Countries that had total COVID-19 vaccination rates of less than 20% were Malawi (15%), Burkina Faso (12.1%), Mali (10%), Senegal (8.8%) and Cameroon (5.9%). The mean, full partial and total vaccination rates for the 20 African countries reviewed were 6.6%, 26% and 32.4% respectively (Table S1).

**Figure 3:**
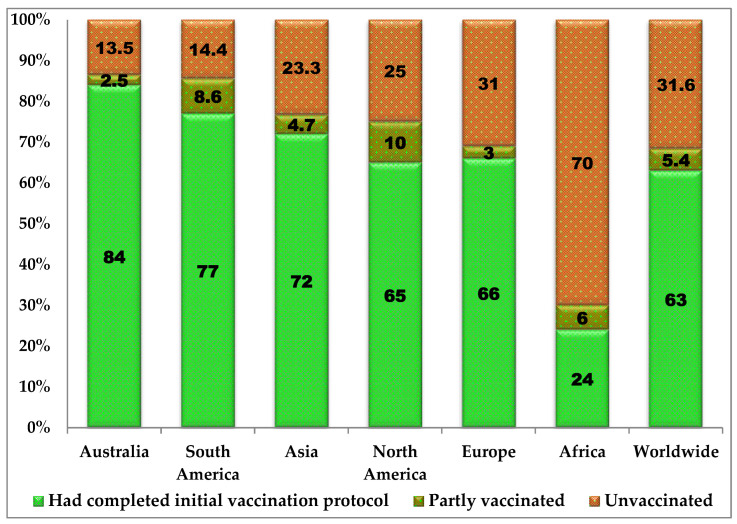
Global distribution of COVID-19 vaccination status in various continents as of 25 October 2022 (adapted from https://ourworldindata.org/coronavirus, accessed on 25 October 2022) [13]).

**Figure 4:**
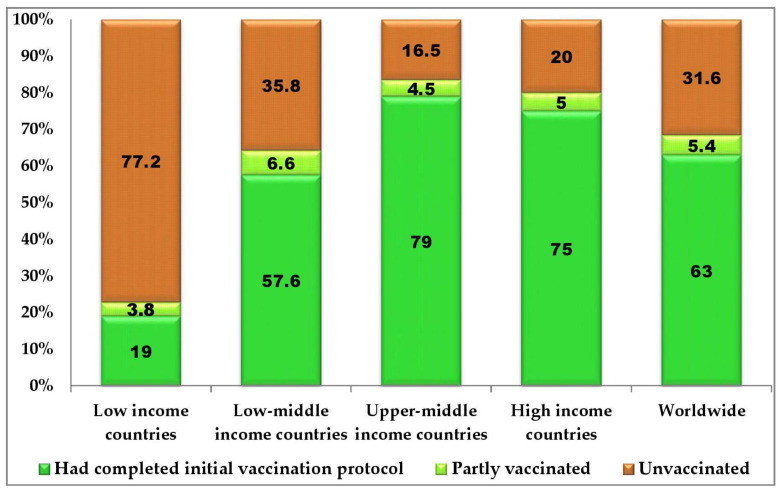
Global distribution of COVID-19 vaccination status according to World Bank’s classification by income levels as of 25 October 2022 (adapted from: https://ourworldindata.org/coronavirus, accessed on 25 October 2022 [13]).

On the continental COVID-19 vaccination status, Africa trails behind the other continents with only 24% of the population fully vaccinated as against the global full vaccination rate of 62% (Figure 3). Australia, South America, Asia, North America and Europe had full COVID-19 vaccination rates of 84%, 77%, 72%, 65% and 66%, respectively (Figure 3). Based on the World Bank’s countries classification of income level, low-income countries had only a 19% full COVID-19 vaccination rate as against the 63% global vaccination rate (Figure 4). Low-middle-income countries, upper-middle-income countries and high-income countries had 57.6%, 79% and 75% full COVID-19 vaccination rates (Figure 4).

### 3.5. Reported Cause of Vaccine Hesitancy in Africa and Ways-Out

As in most other parts of the world, the drivers of VH in Africa are confidence, complacency, convenience, communications, and context. Specifically, safety concerns due to speedy COVID-19 production and roll-out, religious beliefs, lack of trust in the effectiveness of the vaccine, difficulty in the vaccination request/registration protocol and bad feelings towards the vaccines due to negative social media reports were the major causes of VH reported (Table 4). Additionally, COVID-19 vaccine inequality, lack of vaccine production/maintenance facilities, insecurity, high illiteracy level, endemic corruption, mistrust in some political leaders, the spread of unconfirmed anti-vaccination rumors and political instability are the context-specific factors driving COVID-19 VH in Africa. Detail on the causes and suggested solutions to VH as reported in various African countries are presented in Table 4.

## 4. Discussion

### 4.1. Causes of COVID-19 Vaccine Hesitance in Africa

The difference in COVID-19 VARs found (Table 1) reflects the huge diversity in VH across various settings and populations in the African continent. Razai et al. [99] identified major factors (five Cs) responsible for COVID-19 VH to include confidence (safety and efficacy of vaccines), complacency (perception of low risk and low disease severity), convenience (accessibility, easy pre-vaccination protocol), communications (awareness creation, enlightenment campaign) and context (ethnicity, religion and other socio-demographics). Undoubtedly, these five Cs have greatly contributed to COVID-19 VH in Africa. Poor confidence in COVID-19 vaccines due to safety concerns, regarding the hasty production and roll-out, deployment of mRNA vaccine technology and records of serious or life-threatening side effects have been reported as some of the reasons for low COVID-19 VARs in Africa [26,41,51,91]. Similarly, other factors connected to complacency, convenience and communication have also been reported. These include the perception of COVID-19 as “a white man’s disease”, which cannot easily kill the blacks, difficulty in accessing the vaccination due to tough pre-vaccination protocols, spreading of unconfirmed anti-vaccine rumors, infodemic and language barriers during vaccine enlightenment campaigns in rural areas [75,79,80,82,85,94]. The factor of context is even more complex and somewhat peculiar to Africa. Difficult to reach terrain, gender, age, location of residence (urban or rural), and tribal and religious sentiments have also been reported as causes of COVID-19 VH in various parts of the continent [18,21,100].

Africa is deeply religious and faith-based beliefs generally affect VH in the continent. Some faith-based organizations are opposed to vaccination because of the suspicion that some vaccines contain alcohol or pig tissues, and it is religiously wrong to consume these substances [101]. Others are suspicious that some vaccines, particularly COVID-19 vaccines, contain microchips considered to be “the mark of the beast” while some hold that vaccination is a depopulation strategy from the West. These religious dogmas, which are rampant in African countries, may have contributed to low COVID-19 VARs in the continent.

Apart from the five Cs, there are other African-specific determinants of COVID-19 VH. These include vaccine inequality, national income level, lack of vaccine production and maintenance facilities, insecurity, high illiteracy level, endemic corruption, trust deficit, infodemic and political instability. Perhaps, vaccine inequality, especially during the commencement of the COVID-19 vaccine roll-out, is one of the major reasons for the persistent low VARs in Africa. Most African countries commenced COVID-19 vaccination campaigns in the first or second quarter of 2021 while the mass vaccination started in the last quarter of 2020 in some other continents. The low availability of the vaccine amidst the very high global demand and limited production capacities of the few approved COVID-19 vaccine manufacturing companies was partly responsible for the delayed roll-out in Africa. Additionally, it appears that there was no equitable distribution of the available vaccines ab initio. While richer countries practically bought up all available COVID-19 vaccines and even placed advanced orders for yet-to-be produced vaccines [98], low-income countries, most of which are in Africa, were financially constrained in procuring the vaccines for their citizens. Consequently, most African countries received their first batch of COVID-19 vaccines during the first or second quarter of 2021 via the COVAX facility and donations from some countries [102]. Some of the COVID-19 vaccines donated to Africa were alleged to be soon-to-expire vaccines or the vaccine brands rejected by the citizens of the donor countries due to safety concerns [103]. The vaccine inequality, occasioned by disparities in nations’ income levels, and the consequent delayed roll-out may have played some roles in the low COVID-19 VARs in the continent as shown in Figure 3 and Figure 4. While the vaccine inequality lasted, no African countries could commence COVID-19 vaccine manufacturing due to grossly inadequate facilities and technology/man-power for the vaccine production. However, in an attempt to liberalize vaccine manufacturing and boost the production in low- and middle-income countries, the WHO in February 2022 selected and announced that six African countries—Egypt, Kenya, Nigeria, Senegal, South Africa and Tunisia—will receive equipment and training for the production of mRNA vaccines, including COVID-19 vaccines [104]. The selected countries will benefit from the global mRNA technology transfer hub in Cape Town, South Africa, established to scale up vaccine production in low- and middle-income countries.

Although the vaccine inequality has considerably improved in 2022, the acceptance rate in many African countries has ironically reduced instead of increasing (Table 2). This may be due to COVID-19 vaccine politicization, political instability and insecurity (terrorism, banditry and kidnapping for ransom) in some African countries, which could hamper the vaccine administration and acceptance [105]. Between 2020 and 2022, political instability has been on the rise in Africa, with four military coups in Chad, Mali, Guinea Bissau and Sudan and two unsuccessful military takeovers in Niger and Sudan. Furthermore, attacks, kidnappings and killings of vaccine administrators have historically been reported in parts of Africa and Asia [106]. These political and insecurity problems directly and indirectly affect COVID-19 vaccine administration and acceptance, and hence the low VARs reported, even among HCWs as they are being attacked, kidnapped and killed [106].

In addition, people’s mistrust in some political leaders who usually appeal to the general public to get vaccinated has been a major setback to acceptance of COVID-19 vaccination in some African countries [105,107]. The trust deficit may be due to the inability of most political leaderships in Africa to fulfill their political campaigns promises. Shortly after political ascendancy, some of the leaders usually abandoned their electoral promises and programs pledged to the populace during the electioneering period [108]; hence, the massive distrust on politicians in some African countries. Apart from the political class, some Africans, especially in rural settings, also do not trust modern medicines, including COVID-19 vaccines. After the unfortunate death of 11 children in Kano State, Nigeria following a drug trial in 1996 by one of the COVID-19 vaccine manufacturers, some Nigerians still hold that vaccination generally is a strategy by some Western government to depopulate and render Africans sterile [109,110]. The synergy of low adult literacy level, religious bigotry and circulation of unverified anti-vaccine rumors is driving VH in Africa [111,112]. Less-educated people are unlikely to critically appraise the credibility of any anti-vaccine story and may therefore wholeheartedly believe such hearsay and either reject or hesitate to accept COVID-19 vaccines.

Furthermore, corruption, which is endemic in some parts of Africa, has greatly hindered the progression and provision of health-related services, such as COVID-19 vaccination. Public corruption is detrimental to COVID-19 vaccination and acceptance as the vaccine doses could be stolen; funds allocated for public health emergency services could be embezzled through fraudulent procurement systems; and equitable sharing of the vaccine may be distorted via nepotism, favoritism and vaccine nationalization [113]. Vaccines freely donated or procured by the government for administration to the general public may be hoarded for personal financial gains or served only to individuals who are willing to offer bribes [114]. Sometimes, agencies of governments or law enforcement agencies may be aiding this unethical practice.

### 4.2. Global Public Health Implication of Africa’s Low COVID-19 Vaccination Rate

Low COVID-19 vaccination rates facilitate community transmission of SARS-CoV-2 and enhance the development of VOCs, which may be more virulent and transmissible than the parent pathogen [115,116]. Genomic alterations (mutations and deletions) at the RBD of the S1 segment of SARS-CoV-2 spike proteins may affect the spread and infectivity of the SARS-CoV-2 VOCs [116], thereby endangering public health. Consequently, new variants may then spread globally, as has happened in all the VOCs currently described. Genomic recombination events during community transmission of SARS-CoV-2 due to low vaccination coverage or between SARS-like coronaviruses in animals over time may lead to the emergence of the new virus superbug [117]. Recent research evidence indicates that the SARS-CoV-2 Omicron variant may have emerged from a mouse host, due to inter-species SARS-CoV-2 infections, as this variant binds better to mouse ACE-2 receptors than other VOCs [118]. Zoonotic and reverse zoonotic transmissions of SARS-CoV-2 have been reported but human-to-animal transmissions are more common than animal-to-human transmissions [119,120]. Inter-species transmission of SARS-CoV-2 and low COVID-19 vaccination rates are major global public health concerns because these facilitate the virus adaptation in new hosts, which may aid proliferation of more VOCs and their subsequent jumping of species barriers [121]. Each variant may possess enhanced capability for infectivity, transmissibility, pervasiveness and virulence, and these may prolong the global eradication of the pandemic and the resultant public health and economic consequences.

In view of these, low COVID-19 vaccination rates in low- and medium-income countries have enormous global public health significance. For instance, four of the five VOCs so far described emerged from low- or middle-income countries [9], where COVID-19 VARs were sub-optimal, but the virus rapidly spread globally due to their enhanced transmissibility and infectivity potentials. The continued development and global spread of SARS-CoV-2 VOCs, as a result of the low regional vaccination rates against the virus, portends great danger for global public health safety as all the recent gains towards the eradication of the virus could be reversed or at least slowed down. This is particularly true for the SARS-CoV-2 Omicron variant and the sub-variants, which are currently driving the pandemic and have been reported to evade COVID-19-vaccine-induced immunity, despite recent vaccination [122]. Although COVID-19 vaccination significantly reduces the mortality and severity of the disease [123], the immune invading potentials of the new variants make the global population naive and susceptible to the new virus superbug. This is an enormous public health problem as even individuals that were fully vaccinated against COVID-19 may need to take additional doses of the vaccine, to boost their immunity.

### 4.3. The Ways-Out

To reduce VH in Africa, particularly COVID-19 VH, it is imperative to improve both the vaccine availability and acceptance. The problem of vaccine availability in Africa, and indeed in all low- and low-middle-income countries, can only be addressed if the root-causes of vaccine inequality are confronted frontally. For ages, most African and low-income countries have been largely dependent on vaccines produced or donated from richer and more technologically advanced countries for their vaccination needs. Only very few, if any, African countries can boast of self-sufficiency in vaccine production and delivery for the prevention and control of VPDs that are endemic in their localities. This is largely due to the huge infrastructural deficit in vaccine production and preservation logistics in Africa; inadequate technocrats in vaccine research and manufacturing; and the reluctance of some technologically advanced vaccine-producing companies to share their technologies and experiences. Rather than overdependence on other countries for vaccine supply, various national governments in African should invest massively in the health-research sector, especially in the field of vaccinology. They should also cash into the WHO-facilitated global mRNA technology transfer hub in South Africa, designed and being implemented to boost mRNA vaccine production in low- and middle-income countries. Moribund vaccine production facilities, scattered across the continent, should be resuscitated and revamped. Regular staff training and re-training on modern vaccine production and maintenance protocols should be provided to vaccine researchers and HCWs. Although they deserve their patency rights, vaccine producers in high-income and technologically advanced countries should be magnanimous enough to share their vaccine-production technologies with developing countries, at least for global public health safety. These could boost the availability of vaccines, including COVID-19 vaccines, for the control of VPDs in Africa and significantly decrease the emergence of VOCs and the subsequent global spread.

When sufficient COVID-19 vaccines doses are made available in Africa, the next challenge is to improve the vaccination rate by addressing the fundamental causes of VH, which could sometimes be context-specific. Building confidence in the safety and efficacy of vaccines, especially COVID-19 vaccines, in Africa could be achieved by enhanced public enlightenment campaigns and effective communication through strategic, targeted, robust, unambiguous and transparent information dissemination at the national, state and local-government levels. Since there is mistrust in some public office holders, religious and traditional leaders, who still enjoy public trust and have enormous influence on the general public in Africa, could lead the public engagement and education on the dangers of VH. In the African context, these leaders are highly respected and therefore stand the better chance of persuading their followership to drop the fallacy of “COVID-19 being a white man’s disease” and to accept the vaccination, much more than political leaders. In the same vein, non-governmental organizations and multinational companies (as part of their corporate social responsibility) could lead an all-round advocacy campaign against COVID-19 VH, especially in rural areas. Scientific proofs on the safety and efficacy of COVID-19 vaccines, particularly the mRNA-based vaccines, should be shared with the public and translated into local languages to allay public fears regarding the safety of the vaccines. These could help counter the infodemic and unconfirmed anti-vaccination rumors spreading in the society, build public trust on COVID-19 vaccines and enhance the acceptance rate for global public health safety. As in the oral polio vaccination, a house-to-house COVID-19 vaccination campaign could be adopted in Africa and all forms of pre-vaccination bureaucratic bottleneck dismantled to ease the vaccine accessibility and increase the acceptance rate in the continent.

On the African-context-specific COVID-19 vaccination barriers, various national and regional African governments should take decisive steps to ensure that public/institutional corruption, political instability, insecurity, ethnicity, religion and other socio-demographics bias do not continue to hamper COVID-19 vaccine acceptance in the continent. To this end, those who are culpable of stealing or hoarding vaccines or embezzling funds meant for public health services should be publicly reprimanded to deter others from doing same. Security should be beefed up especially around vaccinators and vaccination centers to limit attacks, kidnapping for ransom and killings of health personnel and patients, as this could discourage people from coming out to be vaccinated. Religious leaders should work towards re-orientation of people who holds extreme religious views against vaccines and vaccinations generally. The 67.4% 2021 adult literacy level in Africa, which lags behind the 90% global average [124], needs to be improved as uneducated individuals are more likely to be vaccine-hesitant and peddle anti-vaccination rumors [125]. As the production and availability of COVID-19 vaccines continues to improve, the vaccination of pets and zoo-animals from which reverse zoonotic transmission of SARS-CoV-2 have been reported could be considered. This could limit the evolution and spread of new VOCs and also prevent possible SARS-CoV-2 epizootic or panzootic diseases in susceptible animal species.

## 5. Conclusions

With an overall mean COVID-19 acceptance rate of 58 ± 2.4%, VH still subsists in Africa. On a regional level, the highest mean COVID-19 VARs of 83.5% and 68.9% were recorded in Southern Africa and Eastern Africa, respectively, while Northern Africa had the lowest mean acceptance of 45.1% in 2021. The regional trend in COVID-19 VARs in 2022 was largely the same, with Southern and Eastern Africa maintaining their leads while Central Africa had the lowest acceptance rate of 13.5%. The reduction in the overall annual mean acceptance rates of 59.8 ± 3.8% and 56.6 ± 3.2% in 2021 and 2022, respectively, suggests worsening COVID-19 VH in the continent. Only 23% of Africans have had an initial two-dose COVID-19 vaccination protocol compared to 84%, 79% and 62% reported, respectively, in Australia, upper-middle-income countries and globally. Apart from the problems of confidence, complacency, convenience, communications and context, which are already known determinants of VH globally, some specific factors, including vaccine inequality, low national income level, lack of vaccine production and maintenance facilities, insecurity, high adult illiteracy level, endemic corruption, trust deficit, circulation of unconfirmed anti-vaccination rumors and political instability, may be responsible for the low COVID-19 vaccination rate in the African continent. The low VARs in Africa have tremendous global public health implication as they may facilitate the emergence of immune invading SARS-CoV-2 VOCs, which may spread worldwide. Consequently, there is a need to confront these challenges squarely and engage traditional and religious leaders in the fight against VH in Africa, to restore public confidence in the safety and efficacy of vaccines generally. Incorporation of the S1 segment of the Omicron variant/sub-variants in the original COVID-19 vaccine to widen the protection spectrum may be worthwhile. As the availability of COVID-19 vaccines improves, the vaccination of pets and zoo-animals from which reverse zoonotic transmission of SARS-CoV-2 have been reported could be considered to limit the evolution and spread of new VOCs and avert possible SARS-CoV-2 epizootic or panzootic diseases in susceptible animal species.

### Limitations of the Study

This paper interpreted COVID-19 vaccine acceptance to mean both the actual vaccine uptake and the vaccination intention, during the selection of the 74 papers included in this review. Although the PRISMA guideline and not the AMSTAR-2 checklist was used in the methodology, the authors critically analyzed the 74 studies reviewed and ensured that papers with possible bias such as inconsistent or incomplete data/results were not included.

## Figures and Tables

**Table 1:** Reported COVID-19 vaccine acceptance rates in African countries between 1 January 2020 and 5 September 2022.

Country	Year of Publication	Target Population	Sample Size	* Acceptance Rate Reported (%)	Study/Reference
Nigeria	2021	Other population	589	29	Reuben et al. [25]
Ethiopia	2021	Healthcare workers	418	54.1	Aemro et al. [26]
Somalia	2021	Healthcare workers	4543	76.8	Ahmed et al. [27]
Ethiopia	2021	Healthcare workers	405	48.4	Angelo et al. [28]
Ethiopia	2021	Other population	2654	97.9	Kanyanda et al. [29]
Ethiopia	2021	Healthcare workers	614	39.7	Mohammed et al. [30]
Egypt	2021	Other population	871	88	Elgendy et al. [31]
Egypt	2021	Healthcare workers	308	26	El-sokkary et al. [32]
Egypt	2021	Healthcare workers	385	21	Fares et al. [33]
Tunisia	2021	Healthcare workers	398	58	El-Kefi et al. [34]
Morocco	2021	Other population	1272	26.9	Khalis et al. [35]
Morocco	2021	Healthcare workers	303	62	Khalis et al. [36]
Egypt	2021	Other population	1011	25	Omar et al. [37]
Egypt	2021	Healthcare workers	2133	54	Saied et al. [38]
South Africa	2021	Healthcare workers	1308	90.1	Adeniyi et al. [39]
Malawi	2021	Other population	1542	82.7	Kanyanda et al. [29]
South Africa	2021	Other population	4440	70.8	Kollamparambil et al. [40]
Zimbabwe	2021	Other population	551	55.7	Mcabee et al. [41]
South Africa	2021	Healthcare workers	1015	89.5	Oduwole et al. [42]
Kenya	2021	Other population	963	96	Carpio et al. [43]
Uganda	2021	Other population	1067	53.6	Echoru et al. [44]
Uganda	2021	Other population	2106	84.5	Kanyanda et al. [29]
Nigeria	2021	Healthcare workers	509	37.7	Isah et al. [45]
Ghana	2021	Other population	2345	51	Acheampong et al. [46]
Nigeria	2021	Other population	517	74.5	Adebisi et al. [47]
Ghana	2021	Healthcare workers	1605	70	Alhassan et al. [48]
Nigeria	2021	Healthcare workers	422	49.5	Amuzie et al. [49]
Nigeria	2021	Other population	886	70	Habib et al. [50]
Burkina Faso	2021	Other population	1742	79.5	Kanyanda et al. [29]
Mali	2021	Other population	1591	64.5	Kanyanda et al. [29]
Nigeria	2021	Other population	1703	86.2	Kanyanda et al. [29]
Nigeria	2021	Other population	440	40	Mustapha et al. [51]
Nigeria	2021	Other population	689	71.1	Okafor et al. [52]
Nigeria	2021	Other population	349	34.7	Uzochukwu et al. [53]
Ghana	2021	Other population	1560	35.3	Yeboah et al. [54]
Zambia	2022	Other population	2400	66	Carcelen et al. [55]
South Africa	2022	Other population	395	59	Wiysonge et al. [56]
Ghana	2022	Other population	108	59.3	Botwe et al. [57]
Cameroon	2022	Other population	591	13.5	Ajonina-ekoti et al. [58]
Ethiopia	2022	Healthcare workers	461	84.4	Abay et al. [59]
Ethiopia	2022	Healthcare workers	404	64	Adane et al. [60]
Ethiopia	2022	Other population	350	18.5	Aynalem et al. [61]
Ethiopia	2022	Healthcare workers	403	61.5	Berhe et al. [62]
Ethiopia	2022	Healthcare workers	319	72.7	Boche et al. [63]
Ethiopia	2022	Other population	422	80.9	Dereje et al. [64]
Ethiopia	2022	Healthcare workers	420	58.8	Mose et al. [65]
Ethiopia	2022	Other population	2317	88	Strupat et al. [66]
Ethiopia	2022	Healthcare workers	191	65.4	Tolossa et al. [67]
Ethiopia	2022	Healthcare workers	1314	74.8	Yilma et al. [68]
Sudan	2022	Healthcare workers	217	55.8	Raja et al. [69]
Egypt	2022	Other population	1053	31.5	Salem et al. [70]
Sudan	2022	Healthcare workers	400	63.8	Yassin et al. [71]
Tunisia	2022	Healthcare workers	493	48.1	Zammit et al. [72]
South Africa	2022	Other population	10,465	40	Engelbrecht et al. [73]
South Africa	2022	Other population	213	57	Govere-Hwenje et al. [74]
South Africa	2022	Other population	1662	50.4	Kahn et al. [75]
South Africa	2022	Other population	2364	83.3	Modi et al. [76]
Malawi	2022	Healthcare workers	400	82.5	Moucheraud et al. [77]
Zambia	2022	Other population	677	33.4	Mudenda et al. [78]
Zimbabwe	2022	Other population	1168	49.9	Mundagowa et al. [79]
South Africa	2022	Other population	1193	68	Katoto et al. [80]
South Africa	2022	Other population	5629	70.8	Burger et al. [81]
South Africa	2022	Other population	5862	76.1	Burger et al. [81]
Botswana	2022	Other population	5300	73.4	Tlale et al. [82]
Tanzania	2022	Other population	232	36.2	Chilongola et al. [83]
Kenya	2022	Other population	665	42	Osur et al. [84]
Nigeria	2022	Other population	1058	80.9	Adedeji-adenola et al. [85]
Nigeria	2022	Other population	3076	50.7	Al-mustapha et al. [86]
Senegal	2022	Other population	607	54.3	Ba et al. [87]
Nigeria	2022	Other population	1283	8.2	Ekowo et al. [88]
Nigeria	2022	Other population	400	69.5	Ajibola et al. [89]
Ghana	2022	Other population	415	73.3	Kyei-arthur et al. [90]
Nigeria	2022	Other population	1525	29	Njoga et al. [91]
Nigeria	2022	Healthcare workers	10,184	92	Nomhwange et al. [92]
Nigeria	2022	Healthcare workers	830	38.8	Nri-ezedi et al. [93]
Ghana	2022	Other population	362	62.7	Okai and Abekah-Nkrumah, [94]
Nigeria	2022	Healthcare workers	420	11.4	Onuminya and Onuminya, [95]
Nigeria	2022	Other population	73	74	Osuagwu et al. [96]
Nigeria	2022	Healthcare workers	305	38.3	Adebowale et al. [97]
Nigeria	2022	Other population	800	34.5	Soyannwo et al. [98]

* Acceptance rate = actual vaccine uptake and vaccination intention.

**Table 2:** Yearly and regional distributions of the overall reported COVID-19 vaccine acceptance rates in Africa between 2021 and 2022.

Year of Study	African Region	Number of Studies Found	Number of Respondents per Survey			* COVID-19 Vaccine Acceptance Rate		
			Minimum	Maximum	Mean ± SD	Minimum	Maximum	Mean ± SEM
2021								
	East	10	405	4543	1486 ± 1311	39.7	97.9	68.9 ± 6.6
	North	8	303	2133	835 ± 640	21.0	88.0	45.1 ± 8.5
	South	3	1015	4440	2254 ± 1096	70.8	90.1	83.5 ± 6.3
	West	14	349	2345	1067 ± 659	29.0	86.2	56.6 ± 5.1
	**Total**	**35**	**303**	**4543**	**1235 ± 1038**	**21.0**	**97.9**	**59.8 ± 3.8**
2022								
	Central	1	591	591	591	13.5	13.5	13.5
	East	16	191	2400	758 ± 696	18.5	88.0	61.2 ± 5.1
	North	4	217	1053	541 ± 360	31.5	63.8	49.8 ± 6.9
	South	9	213	10,465	3675 ± 3391	40.0	83.3	64.2 ± 4.6
	West	15	73	10,184	1430 ± 2536	8.2	92.0	51.8 ± 6.4
	Total	45	73	10,465	1522 ± 2329	8.2	92.0	56.6 ± 3.2
	**Grand total**	**80**	**73**	**10,465**	**1397 ± 1872**	**8.2**	**97.9**	**58.0 ± 2.4**

* COVID-19 acceptance rate = actual vaccine uptake and vaccination intention, SD = standard deviation, SEM = standard error of the mean.

**Table 3:** Yearly and regional distributions of reported COVID-19 vaccine acceptance rates in Africa according to population types—healthcare workers and other populations (non-healthcare workers).

Year of Study	African Region	* COVID-19 Vaccine Acceptance Rates Reported			
		Healthcare Workers		Other Populations	
		Number of Studies Found	Mean ± SEM	Number of Studies Found	Mean ± SEM
2021					
	Central	-	-	-	-
	Eastern	4	54.8 ± 7.9	6	78.4 ± 7.9
	North	5	44.2 ± 8.6	3	46.6 ± 20.7
	Southern	2	89.8 ± 0.3	1	70.8.7 ± 0
	West	3	52.4 ± 9.4	11	57.8 ± 6.2
	**Total**	**14**	**55.5 ± 5.6**	**21**	**62.7 ± 5.2**
2022					
	Central	-	-	1	13.5 ± 0
	Eastern	8	70.5.8 ± 3.4	8	51.9 ± 8.6
	North	3	55.9 ± 4.5	1	31.5 ± 0
	Southern	0	-	9	64.2 ± 4.6
	West	4	45.1 ± 16.9	11	54.2 ± 6.7
	**Total**	**15**	**60.8 ± 5.3**	**30**	**54.5 ± 4.0**
	**Grand total**	**29**	**58.3 ± 3.8**	**51**	**57.9 ± 3.2**

SEM = Standard error of the mean; * COVID-19 acceptance rate = actual vaccine uptake and vaccination intention.

**Table 4:** Major reasons for COVID-19 vaccine hesitancy in some African countries and the suggested ways-out.

Region	Country	Major Reasons for Hesitancy	Suggested Recommendations	References
Central Africa	Cameroon	Lack of confidence in COVID-19 vaccines Discriminatory COVID-19 vaccine distribution patterns in other parts of the world relative to Africa and improper COVID-19 vaccine approval timeline	Debunk myth and address concern for safety and efficacy Equitable distribution of available COVID-19 vaccines to all countries as a global good	Ajonina-ekoti et al. [58]
Eastern Africa	Ethiopia	Rumors on the content of the vaccine Concern about safety, effectiveness and adverse effects—teratogenicity Infodemic or disinformation of the public on the safety and efficacy of the vaccine Lack of adequate information about the vaccine Believe that the vaccine may be a biological weapon Concerns about the safety of vaccines due to the speed of the production	Education on side-effects and the importance of the vaccine to address trust deficit Health sector managers should stress awareness creation to alleviate misinformation Provision of clear information about COVID-19 and the vaccine side effects The public should rely on mass media platforms rather than social media Address safety concerns Continuous communication and health education Awareness creation on the safety and adverse effects Communications and training focusing on young health workers Vaccine literacy addressing misconception Targeted information, sensitisation and engagement campaigns bolstering confidence in the safety of approved vaccines	Aynalem et al. [61]; Mohammed et al. [30]; Angelo et al. [28]; Aemro et al. [26]; Mose et al. [65]; Berhe et al. [62]; Adane et al. [60]; Tolossa et al. [67]; Boche et al. [63]; Yilma et al. [68]; Dereje et al. [64]; Abay et al. [59]; Strupat et al. [65]; Kanyanda et al. [29]
Eastern Africa	Kenya	Lack of adequate information about the vaccine, concerns around vaccine safety and effectiveness, lack of trust and confidence in the vaccine	Evidence-based engagements	Osur et al. [84]; Carpio et al. [43]
Eastern Africa	Malawi	Exposure to negative information about the vaccine The primary reservations were safety concerns about the vaccine in general and its side effects specifically	Targeted information, sensitisation and engagement campaigns bolstering confidence in the benefits and safety of approved vaccines	Moucheraud et al. [77]; Kanyanda et al. [29]
Eastern Africa	Somalia	Misconceptions about the vaccine	Sensitization of the general public to dispel any misconceptions	Ahmed et al. [27]
Eastern Africa	Tanzania	Concerns on safety and side effects	Education on safety and benefits of the vaccine	Chilongola et al. [83]
Eastern Africa	Uganda	Mistrust among most community members regarding COVID-19 vaccines Safety concerns about the vaccine and its side effects	Targeted information, sensitisation and engagement campaigns bolstering confidence in the safety and effectiveness of approved vaccines	Echoru et al. [44] Kanyanda et al. [29]
Eastern Africa	Zambia	Doubt about the safety and effectiveness of vaccine	Provide accurate information through trusted sources of information on the benefits of the vaccine	Mudenda et al. [78]; Carcelen et al. [55]
Eastern Africa	Zimbabwe	Lack of trust in the government and uncertainty about vaccine effectiveness and safety	Targeted education and communication to address concerns about vaccine safety and country of manufacturer	Mundagowa et al. [79]; Mcabee et al. [41]
Northern Africa	Egypt	Absence of enough clinical trials Religious belief and safety concerns Fear of side effects of the vaccine Lack of trust in effectiveness and safety due to speedy production and roll-out of the vaccine. Lack of adequate information about side effects Fear of the hidden infective virus in the vaccine	Provision of sufficient and accurate information about the available vaccines Public health intervention campaigns to change negative attitudes, as an initial step to build trust Promote vaccine confidence with clear, precise, up-to-date information and involve medical personnel Structured awareness campaigns to offer transparent knowledge about the safety and efficacy of the vaccines and the technology	Fares et al. [33]; Omar et al. [37]; El-sokkary et al. [32]; Salem et al. [70]; Saied et al. [38]; Elgendy et al. [31]
Northern Africa	Morocco	Fear of the likely side effects and concern about the effectiveness of the vaccine	Assurance on effectiveness Awareness on safety and efficacy	Khalis et al. [35]; Khalis et al. [36]
Northern Africa	Sudan	Concerns about vaccine safety and effectiveness The risk of developing acute adverse events	Provide accurate information on vaccine safety and effectiveness Construction of health educational programs and more accurate information distributed and advertised by respective health authorities	Raja et al. [69] Yassin et al. [71]
Northern Africa	Tunisia	Fear of vaccine side effects	Effective national information campaign on safety and efficacy	Zammit et al. [72]; El-kefi et al. [34]
Southern Africa	Botswana	Religious beliefs and safety concerns	Target education towards hesitant population	Tlale et al. [82]
Southern Africa	South Africa	Lack of trust in the government Lack of trust in the safety and effectiveness of the vaccine Concerns about side effects, lack of access to the online vaccine registration platform, distrust of government, belief in conspiracy theories having no monthly income and depending on someone else to make vaccination decision Concerns about side effects, belief in conspiracy theories and speed of production	Easy-to-understand information regarding the safety and broader trust-building measures Increase confidence in vaccine efficacy. Clarify eligibility and ensure access to vaccines at times and places that are convenient Communication strategies reassuring safety and efficacy of COVID-19 vaccines and addressing sources of misinformation Trust-building intervention, intensify awareness to debunk misconception, increase vaccine literacy, awareness on efficacy of the vaccine, vaccination promotion campaigns, reinforce the message of vaccine safety and efficacy	Engelbrecht et al. [73]; Kahn et al. [75]; Govere-Hwenje et al. [74]; Wiysonge et al. [65]; Katoto et al. [80]; Kollamparambil et al. [40]; Burger et al. [81]; Burger et al. [81]; Modi et al. [76]; Oduwole et al. [42]; Adeniyi et al. [39]
Western Africa	Burkina Faso	Safety concerns about the vaccine and its side effects, insecurity and political instability	Targeted information, sensitisation and engagement campaigns to encourage confidence in the safety of the approved vaccines	Kanyanda et al. [29]
Western Africa	Ghana	Fear of vaccine’s side effects, safety concerns, mistrust, uncertainty, spiritual and religious beliefs Lack of confidence in the safety of the vaccines	Persuasion using public, religious and other influential figures, balanced and open discussion to frontally address the safety and religious concerns Public health educational intervention to address safety and side effects, strategy for attitudinal change, targeted and integrated public health education, intensify education on the benefits and side effects of COVID-19 vaccines	Yeboah et al. [54]; Acheampong et al. [64]; Botwe et al. [57]; Okai and Abekah-Nkrumah, [94]; Alhassan et al. [48]; Kyei-arthur et al. [90]
Western Africa	Mali	The primary reservations were safety concerns about the vaccine in general and its side effects specifically	Targeted information, sensitisation and engagement campaigns bolstering confidence in the safety of approved vaccines	Kanyanda et al. [29]
Western Africa	Nigeria	Difficulty in the vaccination request/registration protocols Bad feelings towards the vaccines due to negative social media Reports/rumors, personal ideology/religious beliefs against vaccination Concerned about the serious adverse effects of the vaccine Lack of trust in of the government’s policies Lack of confidence in the efficacy of the vaccine Perception of vaccine as being in trial stage, Insecurity and fear of being killed were scaring people from coming out to be vaccinated especially in insurgency-inflicted areas Corruption in procurement and distribution of the vaccines	Address the concerns of the local people rather than dispelling their concerns as merely superstitious and senseless More awareness, health education in indigenous language, targeted awareness creation Health education and promotion for a right attitude Targeted and appropriately designed advocacy and behavioral-change communication messages Liberalize access to vaccine, targeted education to debunk misconception and promote trust Structured educational programs to address safety concerns Improve government trustworthiness, improve health communication, implementation of formulated policies and strategies Awareness on safety and efficacy, provision of education and relevant information, ensure public trust is earned, intensify awareness and health education, national deployment and vaccination plan must be revised and robust enough to address the misinformation on vaccine safety	Ekowo et al. [88]; Onuminya and Onuminya, [95]; Reuben et al. [25]; Njoga et al. [91]; Soyannwo et al. [98]; Uzochukwu et al. [53]; Isah et al. [45]; Adebowale et al. [97]; Nri-ezedi et al. [93]; Mustapha et al. [51]; Amuzie et al. [49]; Al-mustapha et al. [86]; Ajibola et al. [89]; Habib et al. [50]; Okafor et al. [52]; Osuagwu et al. [96]; Adebisi et al. [47]; Adedeji-adenola et al. [85]; Kanyanda et al. [29]; Nomhwange et al. [92]
Western Africa	Senegal	Misinformation, fear of adverse effects and lack of adequate information from health workers	Promote vaccine confidence and reduce misinformation	Ba et al. [87]

## Data Availability

The data supporting the findings of this study are available on reasonable request from the corresponding author, E.O.N.

## Supplementary Materials

The following supporting information can be downloaded at: https://www.mdpi.com/article/10.3390/vaccines10111934/s1, Table S1: Extracted data on COVID-19 infection, fatality, case fatality and vaccination rates as of 25 October 2022 in the 20 African countries reviewed.
