# Supplementary material for: Persisting Vaccine Hesitancy in Africa: The Whys, Global Public Health Consequences and Ways-Out—COVID-19 Vaccination Acceptance Rates as Case-in-Point
Source: Vaccines (Basel). 2022 Nov 15;10(11):1934. doi: 10.3390/vaccines10111934 (PMC9697713; doi:10.3390/vaccines10111934)
Supplement: Supplementary file 1 [file vaccines-10-01934-s001.zip › vaccines-1973694-supplementary.pdf]

Table S1. Extracted data on COVID-19 infection, fatality, case fatality and vaccination rates as at 25 October 2022 in the 20 African countries reviewed.

Country	Conformed COVID-19 cases*	Confirmed COVID-19 fatality*	Case fatality ratio	COVID-19 vaccination uptake rate (%) **		
				Full vaccination	Partial vaccination	Total vaccination
Cameroon	121,652	1,935	1.6	1.3	4.7	6
Ethiopia	493,885	7,572	1.5	5.3	31	36.3
Somalia	27,223	1,361	5	7	29	36
Egypt	515,645	24,613	4.8	12	36	48
Tunisia	1,146,044	29,257	2.5	21	52	73
Morocco	1,265,396	16,280	1.3	4	63	67
Sudan	63,449	4,964	7.8	4.6	9.9	14.5
South Africa	4,026,068	102,257	2.5	4.9	33	37.9
Zambia	333,644	4,017	1.2	3.2	9.6	12.8
Malawi	88,073	2,683	3.1	4	15	19
Zimbabwe	257,893	5,606	2.2	11	30	41
Botswana	326,344	2,790	0.9	8.2	53	56.2
Kenya	338,749	5,678	1.7	6.5	19	25.5
Uganda	169,396	3,628	2.2	13	28	41
Tanzania	39,679	845	2.2	3	34	37
Nigeria	266,043	3,155	1.2	6	20	26
Ghana	170,573	1,460	0.9	9.4	26	35.4
Burkina Faso	21,631	387	1.8	2	12	14
Mali	32,719	742	2.3	2.4	8.8	11.2
Senegal	88,679	1,968	2.2	2.5	6.8	9.3
Average	489,639	11,060	2	6.6	26	32.4

Sources: * = https://www.worldometers.info/coronavirus/ [21]; ** = https://ourworldindata.org/coronavirus [16]
